# Using machine learning to understand determinants of IUD use in India: Analyses of the National Family Health Surveys (NFHS-4)

**DOI:** 10.1016/j.ssmph.2022.101234

**Published:** 2022-09-29

**Authors:** Arnab K. Dey, Nabamallika Dehingia, Nandita Bhan, Edwin Elizabeth Thomas, Lotus McDougal, Sarah Averbach, Julian McAuley, Abhishek Singh, Anita Raj

**Affiliations:** a Center on Gender Equity and Health, Department of Medicine, University of California San Diego, San Diego, CA, USA; b Joint Doctoral Program-Public Health, San Diego State University and University of California San Diego, San Diego, CA, USA; c Department of Obstetrics, Gynecology and Reproductive Sciences, University of California, San Diego, USA; d Department of Computer Science, School of Engineering, University of California San Diego, San Diego, CA, USA; e Department of Public Health & Mortality Studies, International Institute for Population Sciences, Mumbai, India

**Keywords:** Intra-uterine devices, IUD, Male engagement, Couple dynamics, Family planning, Reproductive health, Machine learning, NFHS, India

## Abstract

Intra-uterine devices (IUDs) are a safe and effective method to delay or space pregnancies and are available for free or at low cost in the Indian public health system; yet, IUD uptake in India remains low. Limited quantitative research using national data has explored factors that may affect IUD use. Machine Learning (ML) techniques allow us to explore determinants of low prevalence behaviors in survey research, such as IUD use. We applied ML to explore the determinants of IUD use in India among married women in the 4th National Family Health Survey (NFHS-4; N = 499,627), which collects data on demographic and health indicators among women of childbearing age. We conducted ML logistic regression (lasso and ridge) and neural network approaches to assess significant determinants and used iterative thematic analysis (ITA) to offer insight into related variable constructs generated from a series of regularized models. We found that couples’ shared family planning (FP) goals were the strongest determinants of IUD use, followed by receipt of FP services and desire for no more children, higher wealth and education, and receipt of maternal and child health services. Findings highlight the importance of male engagement and family planning services for IUD uptake and the need for more targeted efforts to support awareness of IUD as an option for spacing, especially for those of lower SES and with lower access to care.

## Highlights

• Prevalence of IUD use has remained extremely low in India for the past two decade.
• This paper used machine learning models with iterative thematic analysis to explore potential predictors of IUD use among currently married women in India.
• The strongest predictor of IUD use was shared marital family planning goals between the male and female partners.
• Prior access to Family Planning services and couples' desire for no more children were also found to be predictive of IUD use in India.
• Findings also highlight the importance of male engagement and family planning services for IUD uptake.

## 1 Introduction

Despite multi-sectoral efforts to increase universal access to family planning services (Sustainable Development Goal 3.7), the world is not on track to achieving universal coverage of met demand for family planning (Hellwig et al., 2019; TRACK20, 2020; United Nations Department of Economic and Social Affairs, 2015). India shows little to no improvement in the use of modern contraceptives and continues to rely heavily on female sterilization alone, which is commonly used only after the achievement of desired family size and sex composition of children (Bankole & Singh, 1998; Dey, Acharya, et al., 2021; Ewerling et al., 2021; Government of India, Ministry of Health and Family Welfare & International Institute for Population Studies, 2022a). Reversible modern contraceptive use that does occur is most often in the form of condom use rather than more effective reversible contraception such as intra-uterine devices (IUDs), impeding healthy birth spacing and consequently increasing the risk for maternal and infant mortality (Conde-Agudelo et al., 2007, 2012). Most recent national data, from the 5th National Family Health Survey (NFHS-5) conducted in 2019–21, indicates that less than 3% of non-pregnant women of childbearing age report IUD use, a comparable proportion to that seen more than a decade ago (Government of India, Ministry of Health and Family Welfare & International Institute for Population Studies, 2022b, 2007).

The ongoing low prevalence of modern reversible contraceptives in general and IUDs, in particular, is striking, given the continued efforts from the Government of India to expand the range and reach of contraceptive options across the public health system. India has a national family planning strategy focused on increasing access and use with an equity lens. (National Health Portal, 2018). Contraceptive availability has improved via the Family Planning Logistics Management Information System (FP-LMIS), and there is increased awareness of FP services and contraceptive options via comprehensive media campaigns (FP 2030, 2020). The low uptake of IUDs is also surprising, given that they are available for free or at a low cost in India's public health system (Sharma et al., 2001). The issue with IUDs appears to be more of an uptake rather than a continuation concern as we find lower discontinuation and higher satisfaction among IUD users relative to those using other forms of contraception in the country (Allen et al., 2009; Sharma et al., 2001).

Research on factors affecting IUD use in India and elsewhere focuses largely on supply-side aspects such as low availability, training of providers, and quality of service provision (Agha et al., 2011; Bhat & Halli, 1998; Prasad et al., 2018; Sharma et al., 2014), but efforts made to affect these factors have been implemented with no change in IUD uptake at scale (Government of India, Ministry of Health and Family Welfare & International Institute for Population Studies, 2022b, 2017). Studies also identify several demand-side factors that influence the uptake and use of IUDs, including concerns regarding side effects and opposition from spouses and family (Agha et al., 2011; Mishra et al., 2017; Payne et al., 2016; Robinson et al., 2016; Singal et al., 2022; Weston et al., 2012). Additionally, there is growing evidence in family planning that recognizes the importance of demand-side factors related to women's agency and male engagement in family planning (Bankole & Singh, 1998; Bhan & Raj, 2021). However, there is a paucity of research examining whether such issues correlate with IUD use in India. That which exists has relied on smaller samples and found that married women who have experienced reproductive coercion or physical violence from partners are more likely to use IUDs, perhaps because it is a contraceptive more easily hidden from a partner and managed by the woman (Chen et al., 2020; Tomar et al., 2020).

Inadequate research focused on correlates of IUD use in India may, in part, be due to its low prevalence and subsequent inadequate power for analysis. Machine learning (ML) offers an important means of examining IUD use with national data, examining potential correlates using an exploratory rather than hypothesis-driven approach, and allowing for analysis of low prevalence data (Bellinger et al., 2017; Bratić et al., 2018; Daoud et al., 2019; DeGregory et al., 2018; Seligman et al., 2018). By employing algorithms to parse data and allow for multiple iterations of variables in the dataset to learn the optimum model for explaining an outcome, ML can consider a broader array of potential correlates that would not be possible using traditional epidemiologic and demographic analyses (Bellinger et al., 2017; LeCun et al., 2015; Mooney & Pejaver, 2018). Consequently, we can identify meaningful correlates of our outcome that we might not have otherwise considered if limited to variables guided by theory and prior research. To that end, this method is hypothesis-generating rather than hypothesis-driven. The approach can yield variables that may seem random in their correlation to the given outcome. However, when ML includes iterative theme analysis, a process of thematic analysis of generated variables in a series of iterative ML models, the approach can also offer conceptual clarity on variables linked with the outcome (Raj et al., 2020). ML with iterative thematic analysis (ITA) has been effectively used to examine demographic and health data in India and is especially useful in understanding low prevalence phenomena and understudied issues (Dehingia et al., 2022; McDougal et al., 2021; Raj et al., 2020, 2021). However, this method has not yet been applied to study contraceptive practices in general or IUD use specifically.

In this study, we seek to expand our understanding of determinants of IUD use in India by integrating ML models with ITA to identify factors associated with IUD using a wide range of possible variables. We use nationally representative NFHS-4 data from India and adopt an exploratory lens of analysis rather than formulating a hypothesis *a priori*. This approach is helpful in generating hypotheses, which can be particularly useful for understanding an under-studied or low prevalence phenomenon such as IUD use. While hypothesis testing analyses or studies are more common in public health research, hypothesis-generating ML studies can contribute significantly to our understanding of a given outcome by identifying new variables related to that outcome, which may not have been considered *a priori.* Findings can guide both future hypotheses-driven examination of predictors of IUD use in India, as well as programmatic and policy responses to improve recognition and uptake of IUDs as a safe and effective contraceptive option for women in India.

## 2 Methods

### 2.1 Data

We used data from NFHS-4, a nationally representative survey focused on population, health, and nutrition. The survey was conducted from 2015 to 2016 across all states of India. A total of 699,686 women of age 15–49 years were interviewed in this survey, of whom 499,627 were currently married (Government of India, Ministry of Health and Family Welfare & International Institute for Population Studies, 2017). We limited our analytic sample to married women due to the very small number of the 200,059 unmarried women in the sample who reported IUD use (n = 54; 0.03%). The NFHS-4 questionnaire asks a wide variety of questions, including socio-demographic and household characteristics, reproductive and maternal health outcomes, access to reproductive and maternal health services, financial and economic agency, and social outcomes such as freedom of mobility and decision-making power within the household. Please see the NFHS-4 questionnaire for the full list of questions (Government of India, Ministry of Health and Family Welfare & International Institute for Population Studies, 2015).

### 2.2 Measures

The outcome of interest in this analysis is IUD use among currently married women at the time of the survey, including postpartum IUD uptake prior to leaving the hospital after childbirth (PPIUDs). We included all variables in the NFHS-4 dataset as independent variables in our analysis, though they were pre-processed prior to inclusion in three steps. These variables covered respondents' socio-demographic characteristics, marriage and cohabitation, sexual and reproductive health, birth history, fertility preferences, and knowledge and use of contraceptives. The survey also included questions on contacts with community health workers and women's pregnancy, delivery, and postnatal care. Other topics included children's nutrition, immunization status and health, husband's background and woman's work, other health issues including tobacco consumption, items related to HIV/AIDS and other Sexually Transmitted Infections (STIs), and questions on household relations. Interested readers can find the complete list of variables in the Standard Recode Manual for the Demographic and Health Surveys (DHS) (USAID, 2018).

First, two researchers (AKD and ND) reviewed the complete dataset to identify irrelevant, redundant, and endogenous variables. Example irrelevant variables (i.e., variables which have no meaningful information about the respondent) were respondent id, household number, and respondents’ line number. An example redundant variable was age, represented as a categorical and a continuous variable; we only retained the continuous variable. We also excluded endogenous variables, which we viewed as variables synonymous with the outcome, such as ever IUD use or ever contraceptive use. Second, we used the variable in the format or categorization most commonly used in the literature so that we could compare the results of our ML models to results from existing literature. For example, birth parity is often categorized as zero, one, two, and three or more births, and hence the variable on the number of children/live births was categorized as such. Third, the categorical variables were one-hot encoded, converting variables into multiple binary forms or dummy variables (Coates & Ng, 2011). One-hot encoding is a standard practice carried out before the implementation of ML models. Once we completed these steps, we had over 6500 variables in our set of independent variables or potential correlates of IUD use for ML analysis.

### 2.3 Machine learning models

ML approaches are often used to predict values of an outcome Y given a set of input variables X (X = X_1_, X_2_, …X_p_). Supervised ML techniques solve this problem by first using a dataset for which Y and X are both known to estimate a function *f* that captures the relationship between Y and X. This function *f* is then used to predict Y for new or unseen data where X is known, but Y is unknown. While there are both parametric and non-parametric approaches to estimate *f,* we focus on parametric approaches in this paper. The parametric approach first involves making an assumption about the functional form of *f* (for example, *f* is a linear model)*.* The next step involves fitting or training the model using observed data (or training data). Finally, the performance of the model is evaluated on a test dataset. A potential disadvantage of these approaches is that the assumption made about the function *f* in the first step does not match the true form of *f.* When researchers choose a simpler model to approximate a more complex true form of *f*, it leads to errors in the prediction that are referred to as *bias.* When researchers choose more flexible models on training datasets, the models follow errors, or noise, in the data too closely – a phenomenon known as *overfitting*. Such models would thus perform very well on training (or observed) data but not on test (or unseen) datasets. Please see James et al., 2021, for more details on these concepts.

In this paper, we conducted two types of supervised ML models - a) an L1 regularized logistic regression model, or Least Absolute Shrinkage and Selection Operator (Lasso), b) an L2 regularized logistic regression model or ridge, and c) a neural network (NN) model. Lasso regression was used to reduce the number of predictors in the model. Ridge regression was used to take the model with reduced predictors obtained from lasso and reduce overfitting in the model and multicollinearity, given that the dataset is high dimensional. We then used ITA, described below, to generate coherent and relevant results from the selected variables. The NN model was developed as an explorative exercise to validate our findings from the ridge regression and to assess whether accounting for non-linear relationships among the predictors generated different results. Here, we first describe each of these three ML models and then discuss the analytical approach to implementing the models.

#### 2.3.1 Least Absolute Shrinkage and Selection Operator (lasso)

Lasso is a type of regression model used widely as a powerful tool for data reduction or selecting variables (also known as “features” in ML models) in cases where models have many variables (Seligman et al., 2018; Stock & Watson, 2012). Lasso is an example of a regularized ML model, which imposes a penalty on the size of regression coefficients to avoid overfitting in the model. It tries to shrink coefficients towards zero, which helps researchers eliminate predictors from a model (Ng, 2004). Regularized estimators are restricted forms of maximum likelihood estimators (MLE) since they maximize the likelihood function subject to restrictions on the logistic regression parameters. A traditional regression model differs from a regularized model in having an additional regularizing parameter. The log-likelihood function for lasso takes the form:

$$l_{\theta }\left(y|X\right)=\sum_{i}-\log(1+e^{{-X}_{i}\theta })+\sum_{y_{i}=0}-X_{i}\theta -\lambda \left|\theta \right|$$

Where X is the vector of features or variables and θ is the column vector of the regression coefficients. λ is the tuning parameter, and the term λ|θ| is the regularizer, which allows the model to carry out multiple iterations for the log-likelihood function to find the best values for all the betas (coefficients) in the equation while mitigating overfitting and bias. The larger the λ, the stronger its influence is, and fewer non-zero parameters are selected. When λ = 0, the solution is the ordinary MLE. Existing ML methods include several different approaches to choosing the value of λ. We used k-fold cross-validation for our lasso model, a technique that we describe subsequently.

Using the regularization term, the lasso model shrinks the value of coefficients for the variables that are least related to the outcome to an exact zero. For models with a lot of noise or with a lot of redundant variables, the lasso model can thus help identify irrelevant variables by forcing the coefficient values to zero.

#### 2.3.2 L2 regularized logistic regression model (ridge)

Like lasso, the ridge is also a regularized ML model. However, the ridge does not force the coefficient values to exactly zero. It is thus appropriate to use when we have a reduced set of independent variables that are known to be potentially related to the outcome. The log-likelihood function for a ridge model is:

$$l_{\theta }\left(y|X\right)=\sum_{i}-\log(1+e^{{-X}_{i}\theta })+\sum_{y_{i}=0}-X_{i}\theta -\lambda {\left|\left|\theta \right|\right|}_{2}^{2}$$

Like our lasso approach, we selected the tuning parameter λ for ridge using k-fold cross-validation. The coefficient values of variables from the ridge model determined the importance of a variable based on the strength of association with the outcome. Similar to prior studies that have implemented the analytical approach used in our study, we used the knee point or the point of maximum curvature for a graph of the coefficient values to determine the correlated variables of interest (Satopaa et al., 2011). We first sorted the coefficients of all variables from the regression model from high to low. We then plotted these coefficient values and identified the point of maximum curvature (using the *kneed* library in Python). Finally, we extracted relevant variables from the model by selecting those that had coefficient values higher than the knee point or the point where the coefficient curve becomes flat. We do not use p-values to select correlates because p-values generated using ML are very small and, thus, do not offer a conservative selection process.

#### 2.3.3 Neural network (NN) model

We used a neural network model to account for non-linear relationships among the predictors and compared the results from our iterative thematic categorization method. Before implementing a neural network model, we used a lasso model to eliminate unrelated variables from our dataset and allow for a parsimonious input dataset.

NNs are powerful statistical tools that can perform non-linear discriminant analyses and have been increasingly used in social sciences and epidemiology (Duh et al., 1998; Kreatsoulas & Subramanian, 2018; Qian & Sejnowski, 1988; Seligman et al., 2018). While there are several kinds of NNs, we used feed-forward neural networks, where the input travels in one direction; data passes through the input nodes and exits on the output node. The NN is a fully connected set of nodes organized into several layers. Nodes are logical structures composed of two parts; the first part receives incoming information (inputs) from many sources, and the second part mathematically transforms the input into output information (outputs). This model uses variables or features with a non-zero coefficient estimate from lasso as input units and women's use of IUD as the output. Neurons in the NN are arranged in layers that define the successive linkage of inputs and outputs. By increasing the number of layers, the user can increase the functional complexity of the equation relating inputs to the outcome. Additional layers allow us to model higher-order, non-linear associations between the input units and the output. Our model used four hidden layers.

### 2.4 Measuring the performance of ML models

Before implementing the ML models, we split the data into training and test sets. We assigned eighty percent of our sample as *training* data and randomly assigned the remaining 20 percent as *test* data. We used the training data to train the model while reserving the test dataset to provide an unbiased evaluation of the trained model. We predicted the outcome (for each observation) in the test dataset using the trained ML model and compared the predicted values with the actual outcomes of the observations.

To assess the accuracy of our predictive models, we calculated the area under the receiver operating characteristic curve (AUC). The receiver operating characteristic (ROC) curve is a plot of the test true-positive rate (y-axis) against the corresponding false-positive rate (x-axis), i.e., sensitivity against specificity (Hanley & McNeil, 1982). To assess the error rate, we estimated the Balanced Error Rate (BER), which is the arithmetic mean of the misclassification rate in each class (0 and 1 in this study) (Cawley, 2006; Zhao et al., 2013).

### 2.5 K-fold cross-validation

We used k-fold cross-validation to estimate the hyperparameters for the regularized regression models. In this method, we partitioned the training dataset into k subsets of approximately equal size, and one of the subsets became the validation set. We then used the remaining k-1 subsets as training data. We repeated this process k times, each time with a different validation set, and estimated the optimum value of λ that maximizes the cross-validated log-likelihood. We used 5-folds in our models.

### 2.6 Iterative thematic analysis

To guide a content analysis approach to understanding the variables generated by the ridge model of ML, we used an iterative thematic analysis (ITA) approach developed by the authors and used in prior publications using the same dataset (Dehingia et al., 2022; McDougal et al., 2021; Raj et al., 2020, 2021), in which content experts thematically code variables from ML lasso and ridge models iteratively created. We conducted the ridge regression, and then two researchers (AKD and ND) coded the features with coefficient values higher than the graphed knee point to identify relevant themes. For example, researchers might create a theme titled *socioeconomic characteristic* upon seeing wealth, religion, and residence (rural/urban) as a set of variables in the model, or a theme titled *family planning (FP) counseling* if they see variables on receipt of counseling on side-effects of contraceptives and receipt of counseling on different contraceptive methods.

After generating and coding themes from the first model, we identified variables attached to the theme with the highest variance or coefficient value. We dropped these variables and implemented the ML models of lasso and ridge again, followed by the thematic categorization of variables by the coders. We undertook this process until we found no new themes for at least three consecutive iterations or no new variables in an iteration.

We identified a group of variables as a theme when the number of variables within a given theme was at least 5% of the total number of identified variables above the knee point of the coefficient curve. The standard of 5% is based on the standard for ITA, which has been used in prior publications (Dehingia et al., 2022; McDougal et al., 2021; Raj et al., 2020, 2021), and requires at least two variables in each theme. From the different rounds of analysis, we observed that the conservative estimate of 5% allowed the identification of an adequate number of coherent themes in this analysis. Based on this process, a single variable could be included in multiple themes. If a variable was related to multiple themes, it was retained in the model and taken out only when all the corresponding themes were dropped.

We conducted all analyses using Python with the necessary libraries (pandas, scipy, keras, numpy, sklearn, tensorflow) to develop the predictive algorithms.

## 3 Results

Approximately 1.5% of currently married women aged 15–49 years reported using IUDs in India (Table 1). IUD users and non-users had a similar average age (31 years vs. 33 years) but were found to differ by wealth (33% of IUD users vs. 18% of IUD non-users belonged to the highest wealth quintile), urbanicity (38% of IUD users vs. 28% of IUD non-users lived in urban areas), and parity (28% of IUD users vs. 19% of IUD non-users had one child).

**Table 1:** Descriptive statistics for socio-demographic variables for the overall sample (N = 499,627) and across IUD users and non-users.

	Total sample (N = 499,627)	Current IUD users (n = 9500)	Current non-users of IUD (n = 490,127)
Characteristics	% or Mean (Std. dev)	% or Mean (Std. dev)	% or Mean (Std. dev)
Current age	32.88 (8.46)	31.40 (6.75)	32.91 (8.49)
Years of schooling	6.83 (5.19)	9.32 (5.01)	6.78 (5.18)
Wealth index status:			
Poorest	19.84	8.17	20.07
Poorer	21.59	16.43	21.69
Middle	20.70	19.65	20.72
Richer	19.38	22.22	19.33
Richest	18.49	33.52	18.20
Place of residence:			
Urban	27.88	38.21	27.68
Rural	72.12	61.79	72.32
Religion			
Muslim	12.78	11.49	12.81
Hindu	75.95	61.38	76.23
Others	11.27	27.13	10.96
Caste^a^			
Scheduled Caste/Scheduled Tribe	36.83	37.77	35.30
Other Backward Classes	41.54	31.22	40.03
Other castes/General Category	21.08	26.32	20.12
Region of residence			
North	20.00	35.45	19.70
West	8.29	8.09	8.30
South	13.47	8.43	13.57
Northeast	13.20	25.01	12.97
East	18.92	9.17	19.10
Central	26.12	13.85	26.36
Birth parity			
0	10.28	0.42	10.47
1	18.76	28.47	18.57
2	31.93	29.55	31.79
3+	39.02	31.56	39.17

a Scheduled caste/scheduled tribe and other backward classes are socially marginalized groups.

Ridge Regression and Iterative Thematic Analysis Findings: The first round of lasso followed by ridge regression models generated four distinct themes: *shared marital family planning (FP) goals, FP counseling and FP services, fertility and fertility preferences, and contraceptive use* (Table 2). The theme *shared FP goals* included variables that had the maximum coefficient values. Hence, we dropped this theme and performed another iteration of the ML model, per our ITA approach, and identified three new themes: *contraceptive knowledge, socioeconomic status, and maternal and child health (MCH) services.* In this second iteration, we identified women's access to television as another variable with a coefficient value higher than the knee-point. However, we could not categorize this under any theme due to the absence of any other related variables. We performed the third iteration after dropping the theme with maximum coefficient values (family planning services) but found no new variables, thus ending the ITA process. Hence, we identified seven themes using the iterative thematic categorization method associated with IUD use (Table 2). The AUC and BER of the first round were 88% and 22%, respectively. Whereas, for the second round, the estimates were 87% and 23%, respectively.

**Table 2:** Identified predictors for corresponding themes from the iterative thematic categorization exercise for the complete sample.

Shared marital FP goals	FP counseling and FP services	Fertility and fertility preference	Contraceptive use/non-use	Contraceptive knowledge	Socio- economic status	Access to maternal and child health services
1. Both husband and wife want the same number of children	1. Has been told about the side effects of contraceptives	1. Fertility preference: no more	1. Using contraceptives for limiting	1. Knows about the IUD method	1. Is literate	1. Antenatal care check-ups received: ultrasound, weight, BP, abdomen, blood test, urine test
2. Joint decision (husband and wife) making for contraceptive use	2. Has been told about other FP methods	2. Does not want any more children	2. Using contraceptives for spacing	2. Knows about the traditional method of contraception	2. Highest wealth quintile	2. Received ANC at a private health facility
	3. Has been told about how to deal with side effects	3. Women category: fecund	3. Has never used female sterilization		3. Highest education level: Secondary	3. Received polio vaccine for the child
	4. Has accessed contraceptive from govt. clinic/pharmacies				4. Religion: Sikh, Jain, Other (non-Hindu/non-Muslim)	
	5. Has accessed contraceptives from a private clinic					

The NN model identified nine variables that were associated with IUD use and had a coefficient value higher than the knee point of the coefficient curve (Table 3). There was a significant overlap in the variables identified via the first round of the ITA process and the NN model, with these variables focusing on family planning access and use, shared marital decision-making on family planning and fertility, and the desire for no more children. The AUC and BER of the NN model were 99% and 2%, respectively.

**Table 3:** Identified predictors from the neural network model.

1. Has accessed contraceptive from govt. clinic/pharmacies
2. Has accessed contraceptives from a private clinic
3. Has been told about the side effects of contraceptives
4. Both husband and wife want the same number of children
5. Has been told about other FP methods
6. Has been told about how to deal with side effects
7. Fertility preference: no more
8. Does not want any more children
9. Joint decision (husband and wife) making for contraceptive use

## 4 Discussion

Prevalence of IUD use has remained extremely low in India for the past two decades (<3%), and efforts toward improved public health supply and outreach have yielded no change in IUD use (Allen et al., 2009; Government of India, Ministry of Health and Family Welfare & International Institute for Population Studies, 2022a; Sharma et al., 2001).

A better understanding of factors associated with IUD use may offer guidance on how to improve the uptake of this contraceptive. We used ML models with iterative thematic analysis to explore potential predictors of IUD use among currently married women in India and found that the strongest predictor of this outcome was shared marital family planning goals in terms of both contraceptive decision-making and fertility goals. This finding corresponds with a smaller study from Rajasthan, India, that indicates that joint marital decision-making regarding contraceptive use among IUD users is associated with IUD continuation (Singal et al., 2022). However, it is not in line with research from younger married women from Maharashtra, India, which found a trend in the association between IPV and IUD use, or with a state-representative sample of married women aged 15–49 in Uttar Pradesh that found an association between reproductive coercion and IUD use (Chen et al., 2020; Tomar et al., 2020). Our national findings suggest that male engagement and support for IUDs, and likely couple communication, are important to support the uptake of IUDs (Dey, Acharya, et al., 2021).

While our findings additionally support the importance of FP services for IUD use, these appear to be secondary to the critical importance of male engagement and support. This underscores the need for programs and interventions to engage men in gender-equity-focused family planning interventions. A few such interventions have been implemented with young men (Verma et al., 2006) and young married couples (Fleming et al., 2018) and show some success in improving gender equitable attitudes among men. However, there is a need for more interventions to be developed and tested that focus on other aspects, such as couple communication and male support, specifically for the use of IUDs.

An additional finding of greater importance is the association between the desire for no more children and the use of IUDs, which has not been seen in prior research. Likely, *a priori* determination of this variable as a correlate was lacking, as sterilization is the most common contraceptive and the best option for limiting pregnancy. Clarity on the value of IUDs for birth spacing may be an issue in India and one that requires further examination. Prior qualitative research has identified fear of infertility as a myth or misconception of IUD use that impedes its uptake in India, both by couples and by providers (Ghule et al., 2015; Mishra et al., 2017), and this may be linked to the use of IUDs by women who do not wish for another pregnancy. These findings reinforce calls by these prior qualitative research for greater training and support for IUD uptake both among providers and within communities.

Findings from our study are aligned with prior research that documents higher IUD use among wealthier and more educated women as well as those with greater contraceptive knowledge (Bhat & Halli, 1998; Kumar et al., 2018; Singal et al., 2022). This underscores that community-level support may be particularly important for lower-resource communities to increase their IUD uptake.

We also find that supply-side determinants such as access to maternal and child health services and quality of FP counseling are strong predictors of IUD use. Women who report access to a wide range of healthcare services, such as antenatal care during pregnancy and child immunization, may have greater access to or support for the use of IUDs. These factors underscore the need for a comprehensive rather than a siloed approach to healthcare provision. In addition, the identification of FP counseling quality as a predictor highlights the interactions between service providers and women as an important factor in the uptake of IUDs. Our findings suggest that women who were told about other methods of contraceptives, who were told about the side effects of contraceptives, and who were informed about what to do in case they experienced any side effects were more likely to use IUDs. These findings correspond with prior smaller-scale research from India (Dey, Averbach, et al., 2021) and demonstrate that improving the quality of counseling provided to women may play an important role in increasing the uptake of IUDs as a contraceptive option.

## 5 Limitations

Our study has some limitations. Our data rely on cross-sectional survey research, impeding assumptions of causality and potentially yielding social desirability and recall biases in responses. Additionally, our analyses can only be limited to variables available from the NFHS-4, which is not focused heavily on IUD use; hence, relevant variables may be missing from this analysis, such as awareness of others who use IUDs or concerns regarding IUD side effects related to fear of insertion or worries regarding sex. Such variables may provide further insight into our understanding of the uptake of IUDs in India.

There are also some limitations attached to the ML models we used for these analyses. ML models used in the study do not allow us to account for confounding. By design, these models are exploratory and not driven by theory or prior research; while this is appropriate for an exploratory understanding of IUD use in India, hypothesis-driven analysis can provide more theory-based insights. These models do not identify an exhaustive list of predictors of IUD use among the variables available in the dataset. Instead, the findings reflect themes from variables that account for the most variance in the use of IUDs by women. There are several other ML models, such as tree-based methods, that could have potentially performed better than the models we chose for this study. However, we selected these models based on their performance (accuracy and error rates) with datasets that have many predictors (Seligman et al., 2018; Stock & Watson, 2012) and their validity in prior studies in similar settings (Dehingia et al., 2022; McDougal et al., 2021; Raj et al., 2020, 2021). Finally, the depth and contextualization of this work can be enhanced by qualitative research. While our study offers the generalizability of a nationally representative dataset, it lacks a nuanced understanding of why and how women are engaging in IUD use in India, speaking to the need for more mixed methods approaches on this topic, inclusive of qualitative field studies as well as a broader array of quantitative variables.

## 6 Conclusion

Although there is evidence of a substantial increase in the use of modern contraceptive methods over the last few years in India, usage remains highly skewed towards female sterilization and, secondarily, condom use (Government of India, Ministry of Health and Family Welfare & International Institute for Population Studies, 2022a). IUDs offer a safe and effective contraceptive available at low or no cost from India's public health system, but use remains low.

This study uses ML and ITA to explore correlates of IUD use in India with a nationally representative sample of married women to offer insight into this issue.

We find that the strongest predictor of IUD use was couples’ shared family planning goals, suggesting that family planning programs need to expand their focus from women to couples. At the same time, our findings that desire for no more children correlates with IUD use suggests that recognition and utilization of IUDs for purposes of birth spacing may be unclear; FP counseling needs to emphasize IUDs as an effective reversible contraceptive. We also find that quality of counseling and access to maternal and child health services are important determinants of IUD use, highlighting the importance of integrated reproductive and maternal care as well as the quality of care. While the study adopted an exploratory approach to identify correlations of IUD use, it identified associated themes that researchers can use for future hypothesis testing in this important area of work. At the same time, the evidence from this study reinforces the need for male engagement and high-quality reproductive and maternal care as fundamental to support contraceptive options and use, including the use of IUDs in India.

## Authors’ contribution

AKD: Conceptualization, Investigation, Writing - original draft, Writing - review & editing.

ND: Conceptualization, Methodology, Data curation; Formal analysis, Investigation, Writing - original draft, Writing - review & editing.

NB: Investigation, Writing - original draft; Writing - review & editing.

EET: Project administration, Investigation, Writing - original draft.

LM: Methodology, Investigation, Writing - review & editing.

SA: Investigation, Writing - review & editing.

JM: Methodology, Writing - review & editing.

AS: Methodology, Writing - review & editing.

AR: Funding Acquisition, Conceptualization, Methodology, Formal Analysis, Investigation, Writing - review & editing.

## Funding

Funding for this work comes from the 10.13039/100009633Eunice Kennedy Shriver National Institute of Child Health and Human Development, National Institutes of Health, USA (Grant number: R01HD084453) and the 10.13039/100000865Bill and Melinda Gates Foundation (Grant numbers OPP1179208 and INV-002967). The funders had no role in the study design, data collection and analysis, decision to publish, or preparation of the manuscript.

## Ethical statement

Ethical clearance for the National Family Health Survey 2015-16 was received from IIPS's Ethical Review Board. During the survey, interviewers obtained informed consent from each respondent before the interview.

## Declaration of competing interest

The authors have declared that no competing interests exist.

## Figures and Tables

**Table:** 

Variables	Shared marital FP goals	FP services	Fertility	Contraceptive use/non-use	Contraceptive knowledge	Socio- economic status
v621: husband's desire for children: both want same;	X					
v327: last source for users: government clinic/pharmacy;		x				
v632: decision maker for using contraception: joint decision;	X					
v3a02: told about side effects: yes;		X				
v3a05: told about other family planning methods: yes;		X				
v3a04: told how to deal with side effects: yes;		X				
v602: fertility preference: no more;			X			
v605: desire for more children: wants no more;			X			
s321a: have used: female sterilization: no;				x		
v625: exposure (definition 2): fecund *;			X			
v625a: exposure to need for contraception (definition 3): fecund *;			X			
v623: exposure: fecund *;			X			
v624: unmet need: using for limiting;				X		
v626a: unmet need for contraception (definition 3): using for limiting;				X		
v626: unmet need (definition 2): using for limiting;				X		
v626: unmet need (definition 2): using for spacing;				X		
v626a: unmet need for contraception (definition 3): using for spacing;				X		
v624: unmet need: using for spacing;				X		
v327: last source for users by type: private clinic/delivery;		x				
v304_02: knows method IUD: yes;					X	
v155: literacy: able to read whole sentence;						X
* fecund women calculated as - women who are not pregnant, not postpartum amenorrheic, not menopausal, have been continuously married and have not used contraception in the five years preceding the survey and have not had any children in those five years						

**Table:** 

Variables	FP services	Fertility	Contraceptive use/non-use	Contraceptive knowledge	Socio- economic status	Access to maternal and child health services	Access to media
v327: last source for users by type: government clinic/pharmacy;	X						
v3a02: told about side effects: yes;	X						
v3a05: told about other family planning methods: yes;	X						
v3a04: told how to deal with side effects: yes;	X						
v605: desire for more children: wants no more;		X					
v602: fertility preference: no more;		X					
s321a: have used: female sterilization: no;			x				
v625: exposure (definition 2): fecund;		X					
v625a: exposure to need for contraception (definition 3): fecund;		X					
v623: exposure: fecund;		X					
v626a: unmet need for contraception (definition 3): using for limiting;			X				
v624: unmet need: using for limiting;			X				
v626: unmet need (definition 2): using for limiting;			X				
v626a: unmet need for contraception (definition 3): using for spacing;			X				
v626: unmet need (definition 2): using for spacing;			X				
v624: unmet need: using for spacing;			X				
v327: last source for users by type: private clinic/delivery;	X						
v304_02: knows method IUD: yes;				X			
v155: literacy: able to read whole sentence;					X		
v304_09: knows method - Withdrawal: yes;				X			
v190: wealth index: richest;					X		
s220b_01: ultrasound test: yes;						X	
v130: religion: 3:96;					X		
m42e_1: during pregnancy: blood sample taken: yes;						X	
m42c_1: during pregnancy: blood pressure taken: yes;						X	
v106: highest educational level: secondary;					X		
m42d_1: during pregnancy: urine sample taken: yes;						X	
s418e_1: abdomen examined: yes;						X	
m42a_1: during pregnancy: weighed: yes;						X	
s408a_1: pregnancy testing kit used: yes;						X	
h6_1: received polio 2: vaccination date on card;						X	
v159: frequency of watching television: almost every day;							X
m57n_1: antenatal care: other private sect. health facility: no;						X	
m2n_1: prenatal: some care;						X	

## Data Availability

The study uses data from the Demographic Health Surveys which are available to be dowloaded from: https://www.dhsprogram.com/Data/
